# Multi-Biomarker Points and Outcomes in Patients Hospitalized for Heart Failure: Insights From the China PEACE Prospective Heart Failure Study

**DOI:** 10.3389/fcvm.2022.835465

**Published:** 2022-04-07

**Authors:** Guangda He, Lihua Zhang, Xiqian Huo, Qing Wang, Danli Hu, Xinghe Huang, Jinzhuo Ge, Yongjian Wu, Jing Li

**Affiliations:** National Center for Cardiovascular Diseases, National Clinical Research Center for Cardiovascular Diseases, NHC Key Laboratory of Clinical Research for Cardiovascular Medications, State Key Laboratory of Cardiovascular Disease, Fuwai Hospital, Chinese Academy of Medical Sciences and Peking Union Medical College, Beijing, China

**Keywords:** heart failure, biomarker, prognosis, survival, quality of life

## Abstract

**Objective:**

To quantitatively characterize the pattern of systemic impairment reflected by conventional biomarkers and assess how it relates to clinical outcomes and quality of life among patients hospitalized for heart failure (HF).

**Methods:**

Patients hospitalized for HF from 52 hospitals in China were enrolled between 2016 and 2018. They were divided into developing and validating cohorts; the developing cohort was used for calculating the weights of biomarkers and constructing the multi-biomarker panel, while the validating one was used for evaluating the relationship between multi-biomarker points and outcomes. In total, five conventional biomarkers reflecting various pathophysiological processes were included in the panel: N-terminal pro-B type natriuretic peptide, high-sensitivity troponin T, hemoglobin, albumin, and creatinine. The weights of the biomarkers were defined based on their relationship with cardiovascular death, and each patient had a multi-biomarker point ranging from 0 to 12. The primary clinical outcome was cardiovascular death, and the other clinical outcomes included rehospitalization for HF, all-cause death, and all-cause rehospitalization in 1-year. The quality of life was measured using Kansas City Cardiovascular Questionnaire. Multi-variable Cox proportional hazard models were used to assess the risks of clinical outcomes, and generalized linear models were used to evaluate the quality of life.

**Results:**

In total, 4,693 patients hospitalized for HF were included in this analysis; the median (interquartile range, IQR) age was 67 (57–75) years old and 1,763 (37.6%) were female. The median multi-biomarker point was 5 (IQR, 2–6). There were 18.0% of patients in the low point group (<2), 29.4% in the mid-low point group (2–4), 27.8% in the mid-high point group (5–6), and 24.7% in the high point group (>6). Compared with those in the low point group, the patients in the high point group had a significantly excess risk of cardiovascular death (adjusted hazard ratio: 5.69, 95% CI, 3.33–9.70). Furthermore, patients with higher points were also more prone to worse quality of life.

**Conclusion:**

Systemic impairment reflected by abnormal conventional biomarker values was common amongst patients hospitalized for HF and had substantially cumulative adverse influence on clinical outcomes and quality of life.

## Introduction

Heart failure (HF) is a major public health problem affecting an estimated 64.3 million people worldwide, and it is also a life-threatening clinical syndrome requiring urgent and comprehensive management (1). The measurable circulating biomarkers have been demonstrated to intimately associate with clinical outcomes amongst patients with HF (2). In addition, a series of biomarker assays are strongly recommended by international clinical guidelines to predict the long-term prognosis, e.g., N-terminal pro-B type natriuretic peptide (NT-proBNP) and high-sensitivity troponin T (hs-cTNT) (3). Particularly, conventional biomarker assays are relatively simple, convenient, and without the necessity of high-tech equipment or highly educated clinicians, which makes it feasible and cost-effective to identify high-risk patients for further personalized care (4). To tailor targeted therapy and intensive follow-up rationally, an in-depth understanding of conventional biomarkers could be a promising step for risk stratification.

Although existing studies had examined the prognostic value of biomarkers (e.g., NT-proBNP, hs-cTNT, and hemoglobin) in prognosis prediction (3), several problems remain to be elucidated. Firstly, previous analyses mainly focused on individual biomarkers in isolation, and thus far, the description that focuses on the overall pattern of multiple conventional biomarkers reflecting “systemic impairment” and the basic pathophysiological burden is scarce (5, 6). Secondly, the relationship between multi-biomarker pattern and long-term clinical prognosis has not been quantitatively characterized (5, 6), and the relationship between the overall multi-biomarker pattern and post-discharge quality of life in patients with HF remains understudied. Understanding the implication of multi-biomarker patterns and systemic impairment would be beneficial to identify patients at high risk and tailor optimal treatment in the context of a growing population with HF.

Accordingly, using the data from a nationwide prospective cohort study enrolling patients hospitalized for HF, we aimed to (i) characterize the overall patterns of the systemic impairment reflected by conventional biomarkers, (ii) qualitatively and quantitatively evaluate the association between multi-biomarker pattern and clinical outcomes, and (iii) analyze the relationship between multi-biomarker pattern and quality of life.

## Materials and Methods

### Study Design and Participants

The rationale and design of the China Patient-centered Evaluative Assessment of Cardiac Events Prospective Study of Heart Failure (China PEACE 5p-HF Study) had been published (7). Briefly, the China PEACE 5p-HF Study was a national, multicenter prospective, observational cohort study collecting hospitalization and long-term follow-up data of patients hospitalized for HF. From August 2016 to May 2018, 4,907 patients hospitalized for HF from 52 hospitals (48 tertiary and 4 secondary hospitals) located in 20 of 31 Chinese provinces (covering all economic-geographic regions in China) were enrolled. Eligible patients were ≥18-year old, local residents, and hospitalized with a primary diagnosis of *de novo* acute HF or decompensation of chronic HF. All participants provided written informed consents. In the current analysis, patients were excluded if they died during the index hospitalization, were lost to follow-up, lacked baseline blood samples for central assay, or lacked baseline left ventricular ejection fraction (LVEF). To construct the multi-biomarker point panel, patients were divided into developing and validating cohorts by their patient ID numbers (odd number in the developing cohort and even number in the validating cohort). The flow chart of the study cohort development is shown in Supplementary Figure 1, and the numbers of patients enrolled in each center are shown in Supplementary Table 1.

The China PEACE 5p-HF Study was approved by the ethics committees at Fuwai Hospital and collaborating sites. The investigation conforms with the principles outlined in the Declaration of Helsinki. The study was registered at www.clinicaltrials.gov (NCT 02878811).

### Data Collection

The data of medical charts of index hospitalization were uploaded at collaborating sites and centrally abstracted at the national coordinating center. To ensure the quality of abstraction, we used a two-level quality control approach. Local investigators collected data *via* interview using an electronic data collection software, which enabled automatic data quality check.

The data of demographics, smoking status, and quality of life were collected by physicians using a standardized questionnaire through the interview at baseline. LVEF was measured according to the standard echocardiogram protocol by trained local physicians. The data of clinical characteristics, medical history, examination results, and discharge medication were centrally abstracted from the medical charts of the index hospitalization. We only used the data of medication or therapy applied in >1% of patients, while the medications or therapy utilized in ≤1% of patients (including angiotensin receptor neprilysin inhibitor, sodium-dependent glucose transporters 2 inhibitors, implantable cardioverter defibrillator, and cardiac resynchronization therapy) were not included in the current analysis. The blood samples were taken within 48 h of admission for biomarker assays in the central laboratory.

Local investigators interviewed the enrolled patients in person at 1, 6, and 12 months after discharge. The trained staff at the national coordinating center would conduct interviews *via* telephone for patients who did not attend the scheduled in-person interview.

### Variable Definition

Patients were classified into HF with reduced ejection fraction (HFrEF, LVEF < 40%), mildly reduced ejection fraction (HFmrEF, LVEF 40–49%), and preserved ejection fraction (HFpEF, LVEF ≥ 50%). Comorbidities, including hypertension, atrial fibrillation, coronary heart disease (CHD), myocardial infarction (MI), valvular heart disease (VHD), previous HF, stroke, chronic obstructive pulmonary disease (COPD), reduced renal function, and diabetes mellitus (DM), were defined according to medical history, discharge diagnosis, or positive laboratory results; reduced renal function was defined as estimated glomerular filtration rate (eGFR) < 60 ml/L/1.73m^2^; DM was defined as a history of DM or glycosylated hemoglobin A_1*c*_ (HbA_1*c*_) ≥ 6.5%.

### Multi-Biomarker Panel

In total, twelve conventional biomarkers reflecting nine basic pathophysiological states were evaluated: NT-proBNP, hs-cTNT, hemoglobin, albumin, creatinine, alanine aminotransferase (ALT), high-sensitivity C-reactive protein (hsCRP), HbA_1*c*_, total cholesterol (TC), low-density lipoprotein cholesterol (LDL-C), triglycerides (TG), and high-density lipoprotein cholesterol (HDL-C). Considering the practicability in clinical practice, continuous variables of these biomarkers were transferred into dichotomous variables based on clinically meaningful cut-off values and literature reviews. Only hemoglobin and albumin were analyzed in the local laboratory while other biomarkers were analyzed in the central laboratory. The methods of central biomarker assays were shown in Supplementary Data Sheet 1. If the results of the central analysis were not available, we used the results of the local analysis. NT-proBNP ≥ 1400 ng/l indicated myocardial stretch due to volume overload; (8) hs-cTNT > 14 ng/l implicated cardiac injury; (9) hemoglobin < 120 g/l in men or < 110 g/l in women represented anemia; albumin ≤ 35 g/l reflected hypoalbuminemia; (10) creatinine > 133 μmol/l (1.5 mg/dl) represented renal dysfunction; (11) ALT > 40 U/l reflected hepatic dysfunction; (12) hsCRP > 2 mg/l indicated systemic inflammation; (9, 13) HbA_1*c*_ ≥ 6.5% implicated DM; dyslipidemia was defined as TC > 5.2 mmol/l (200 mg/dl), LDL-C > 3.4 mmol/l (130 mg/dL), TG > 1.7 mmol/l (150 mg/dL), or HDL-C < 1.0 mmol/l (40 mg/dl)(14). The missing rates of these biomarkers were shown in Supplementary Table 2.

### Outcome Events and Adjudication

The primary clinical outcome was cardiovascular mortality within 1-year after discharge. The other clinical outcomes included post-discharge rehospitalization for HF, all-cause mortality, and all-cause rehospitalization in 1-year. Cardiovascular mortality included death due to HF progression, stroke, CHD, VHD, arrhythmia, cardiac sudden death, other cardiovascular reasons, or presumed cardiovascular death/unknown reasons (15). The quality of life, as patient-reported outcomes, was measured by a short version of the Kansas City Cardiovascular Questionnaire (KCCQ) score within 6-month after discharge, a score ranging from 0 (worst) to 100 (best) (16, 17).

We ascertained the deaths with the same approach in a prior international clinical trial (18). Deaths were collected from death certificates, interviews of patients’ relatives, or the national database of death causes. Rehospitalizations were determined based on medical records and interviews of patients. The data of outcomes were adjudicated by clinic staff at the national coordinating center (Supplementary Data Sheet 2).

### Statistical Analysis

Frequency (percent) and median (interquartile ranges, IQR) were reported for categorical and continuous variables, respectively. Pearson’s chi-square test was used to compare categorical variables and the Kruskal-Wallis test for continuous variables.

In the developing cohort, we performed an analysis of the association between cardiovascular mortality (primary clinical outcome) and the aforementioned biomarkers with a multi-variable Cox proportional hazard regression model and only included biomarkers related to cardiovascular mortality (*P* < 0.10) in the final multi-biomarker panel (including NT-proBNP, hs-cTnT, hemoglobin, albumin, and creatinine) (Supplementary Table 3). Candidate covariates were selected based on a review of the literature and clinical experience (including age, sex, SBP, heart rate, New York Heart Association [NYHA] class, history of hypertension, atrial fibrillation, CHD, VHD, previous HF, current smoking [yes or no], LVEF subgroups [HFrEF, HFmrEF, HFpEF or missing LVEF], discharge use of angiotensin-converting enzyme inhibitor or angiotensin receptor blockers [ACEI/ARBs], β-blockers, diuretics, and aldosterone antagonists). We also assessed the Pearson’s correlation coefficient between the included biomarkers and there was no collinearity between these biomarkers (Supplementary Table 4). The weights of biomarkers were defined according to the hazard ratio (HR) value of each biomarker in the Cox model (Supplementary Table 5).

In the overall patient population, to characterize the multi-biomarker pattern, we summed and calculated the multi-biomarker point with the weights. Each patient had a multi-biomarker point ranging from 0 to 12, and these patients were furtherly classified into four multi-biomarker groups based on the median (IQR) of multi-biomarker points in the overall patient population (Supplementary Table 6).

Survival analysis was performed in the validating cohort. We presented and compared the clinical outcomes across multi-biomarker groups using the Kaplan-Meier analysis and log-rank tests. The multi-variable Cox models were used to evaluate the relationship between the multi-biomarker group and clinical outcomes. In addition, we calculated the Harrell concordance index (C-index) for the discrimination of the predicting models comprised of multiple and individual biomarkers.

Furthermore, in the validating cohort, we developed generalized linear regression models to evaluate the relationship between the multi-biomarker group and the KCCQ score. In addition, we only included patients who had KCCQ scores both at baseline and 6-month in the analysis of life quality (Supplementary Figure 1).

We performed a subgroup analysis of the association between outcomes and multi-biomarker points among patients with HFrEF, HFmrEF, and HFpEF as well. For sensitivity, firstly, we developed competing risk models to re-evaluate the relationship between the multi-biomarker group and clinical outcomes. Secondly, to minimize the selection bias caused by the missing data of life quality at the 6-month interview, we performed inverse probability weighting (IPW) analysis to measure the probability of being observed. Thirdly, we also analyzed the relationship between outcomes and multi-biomarker points calculated using beta values.

The missing rate of patient characteristics or biomarkers ranged from 0 to 5.8% (LVEF). All missing variables were inputted with multiple imputation methods with 10 imputations. A two-sided P-value of < 0.05 was considered statistically significant, and all analyses were performed using SAS statistical software version 9.4 (9.4, SAS Institute, Cary, North Carolina) and R software version 3.6.2 (R Foundation for Statistical Computing, Vienna, Austria).

## Results

### Baseline Characteristics by Multi-Biomarker Groups

**FIGURE 1:**
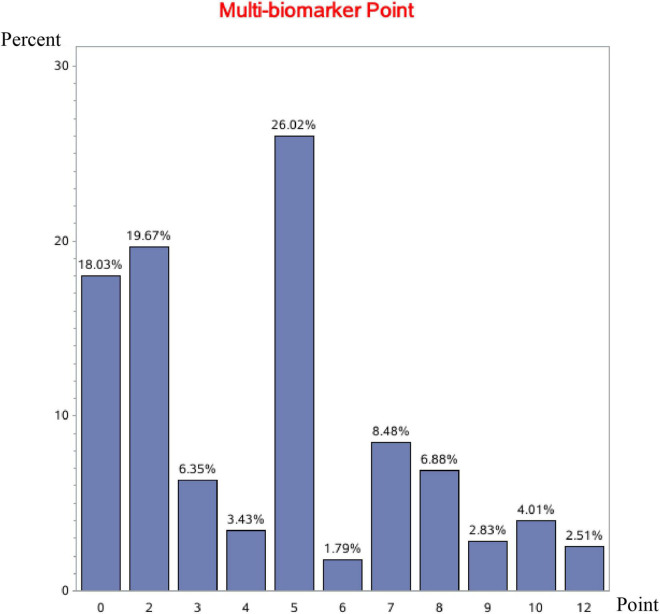
Multi-biomarker point distribution in the overall cohort.

In total, 4,693 patients hospitalized for HF were included in the current analysis. The median age was 67 (IQR, 57–75) years old, and 1,763 (37.6%) were female. The median multi-biomarker point was 5 (IQR, 2–6), and the distributions of multi-biomarker points in overall, developing, and validating cohorts were shown in Figure 1 and Supplementary Figures 2, 3, respectively. In general, there were 846 (18.0%) patients in the low point group (<2), 1,382 (29.4%) in the mid-low point group (2–4), 1,305 (27.8%) in the mid-high point group (5–6), and 1,160 (24.7%) in the high point group (>6).

The baseline characteristics by stratified multi-biomarker groups in the overall population were shown in Table 1. Patients with higher points were older, more likely to be male, and with higher levels of NYHA class. Hypertension, CHD, MI, previous HF, reduced renal function, and DM were more prevalent among patients with higher points. Furthermore, as for discharge medication, patients in the higher point group were less often prescribed ACEI/ARBs and β-blockers. The baseline characteristics in developing and validating cohorts were respectively presented in Supplementary Tables 7, 8.

**TABLE 1:** Baseline characteristics by multi-biomarker point groups in overall cohort.

	Total (*n* = 4693)	<2 points (*n* = 846)	2–4 points (*n* = 1382)	5–6 points (*n* = 1305)	>6 points (*n* = 1160)	*p* value
**Demographic**						
Age, year, median (IQR)	67 (57, 75)	64 (55, 73)	66 (55, 75)	67 (57, 75)	70 (61, 78)	<0.0001
Female, *n* (%)	1763 (37.6)	413 (48.8)	471 (34.1)	480 (36.8)	399 (34.4)	<0.0001
**Clinical characteristics**						
SBP, mmHg, median (IQR)	130 (117, 149)	130 (120, 145)	130 (119, 150)	130 (113, 145)	131 (115, 151)	0.0008
DBP, mmHg, median (IQR)	80 (70, 90)	80 (70, 90)	80 (70, 90)	80 (70, 90)	80 (69, 90)	0.0020
HR, b.p.m, median (IQR)	86 (74, 100)	80 (70, 96)	85 (73, 100)	89 (76, 105)	88 (76, 100)	<0.0001
NYHA class, *n* (%)						<0.0001
III	2076 (44.2)	414 (48.9)	625 (45.2)	566 (43.4)	471 (40.6)	
IV	1932 (41.2)	187 (22.1)	507 (36.7)	620 (47.5)	618 (53.3)	
**Medical history and risk factor**						
Hypertension, *n* (%)	2750 (58.6)	461 (54.5)	830 (60.1)	706 (54.1)	753 (64.9)	<0.0001
Atrial fibrillation, *n* (%)	1712 (36.5)	333 (39.4)	501 (36.3)	501 (38.4)	377 (32.5)	0.0045
CHD, *n* (%)	2715 (58.9)	454 (53.7)	815 (59.0)	700 (53.6)	746 (64.3)	<0.0001
MI, *n* (%)	1075 (22.9)	139 (16.4)	318 (23.0)	288 (22.1)	330 (28.5)	<0.0001
VHD, *n* (%)	759 (16.2)	138 (16.3)	213 (15.4)	231 (17.7)	177 (15.3)	0.3124
Previous HF, *n* (%)	3297 (70.3)	548 (64.8)	967 (70.0)	943 (72.3)	839 (72.3)	0.0007
Stroke, *n* (%)	963 (20.5)	166 (19.6)	274 (19.8)	251 (19.2)	272 (23.5)	0.0411
COPD, *n* (%)	928 (19.8)	172 (20.3)	292 (21.1)	256 (19.6)	208 (17.9)	0.2339
Reduced renal function, *n* (%)	1345 (28.7)	76 (9.0)	289 (20.9)	315 (24.1)	665 (57.3)	<0.0001
DM, *n* (%)	1484 (31.6)	208 (24.6)	429 (31.0)	386 (29.6)	461 (39.7)	<0.0001
Current smoking, n (%)	1178 (25.1)	211 (24.9)	374 (27.1)	351 (26.9)	242 (20.9)	0.0010
**Echocardiograph**						
LVEF,%, median (IQR)	44 (34, 56)	51 (40, 62)	44 (35, 57)	40 (30, 50)	42 (33, 53)	<0.0001
HF phenotype						<0.0001
HFrEF, *n* (%)	1826 (38.9)	206 (24.4)	509 (36.8)	635 (48.7)	476 (41.0)	
HFmrEF, *n* (%)	1144 (24.4)	188 (22.2)	339 (24.5)	321 (24.6)	296 (25.5)	
HFpEF, *n* (%)	1723 (36.7)	452 (53.4)	534 (38.6)	349 (26.7)	388 (33.5)	
**In-hospital treatment**						
Coronary angiography	631 (13.5)	136 (16.1)	219 (15.9)	172 (13.2)	104 (9.0)	<0.0001
PCI	211 (4.5)	30 (3.6)	77 (5.6)	51 (3.9)	53 (4.6)	0.0871
**Treatment at discharge**						
ACEI/ARBs, *n* (%)	2455 (52.3)	460 (54.4)	757 (54.8)	708 (54.3)	530 (45.7)	< 0.0001
β-blockers, *n* (%)	2768 (59.0)	528 (62.4)	838 (60.6)	767 (58.8)	635 (54.7)	0.0025
Aldosterone antagonists, *n* (%)	2973 (63.4)	488 (57.7)	854 (61.8)	909 (69.7)	722 (62.2)	<0.0001
CCBs, *n* (%)	686 (14.6)	145 (17.1)	200 (14.5)	122 (9.4)	219 (18.9)	<0.0001
Diuretics, *n* (%)	3225 (68.7)	521 (61.6)	917 (66.4)	958 (73.4)	829 (71.5)	< 0.0001

*IQR, interquartile range; SBP, systolic blood pressure; DBP, diastolic blood pressure; HR, heart rate; NYHA class, New York Heart Association class; CHD, coronary heart disease; MI, myocardial infarction; VHD, valvular heart disease; HF, heart failure; COPD, chronic obstructive pulmonary disease; Reduced renal function, eGFR < 60 ml/L/1.73m^2^; DM, diabetes mellitus; LVEF, left ventricular ejection fraction; HFrEF, heart failure with reduced ejection fraction; HFmrEF, heart failure with mildly reduced ejection fraction; HFpEF, heart failure with preserved ejection fraction; PCI, percutaneous coronary intervention; ACEI, angiotensin-converting enzyme inhibitor; ARB, angiotensin receptor blockers; CCB, calcium channel blocker.*

### Multi-Biomarker Distribution by Left Ventricular Ejection Fraction Subgroups

A total of 1826 (38.9%) patients had HFrEF, 1144 (24.3%) had HFmrEF, and 1723 (36.7%) had HFpEF (Supplementary Table 9). Patients with HFpEF had significantly lower levels of NT-proBNP and hs-cTNT. The lowest median multi-biomarker point was in HFpEF [3 (IQR, 0-5)], followed by HFmrEF [5 (IQR, 2-7)] and HFrEF [5 (IQR, 2-7)] (P < 0.0001).

### Association Between Multi-Biomarker Point and Clinical Outcomes

**FIGURE 2:**
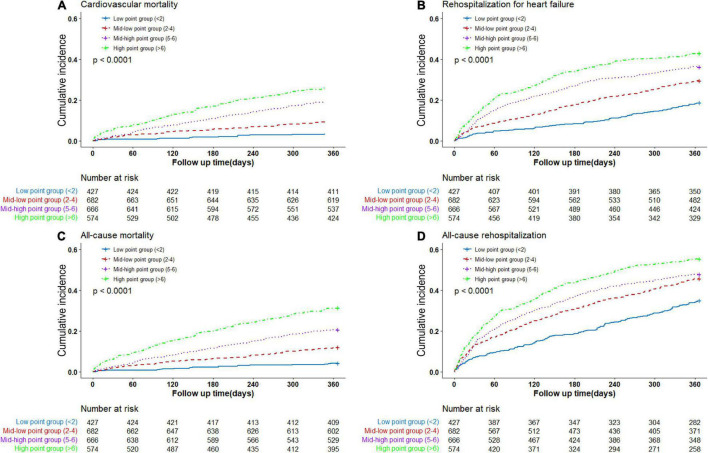
Cumulative event rates by multi-biomarker groups (validating cohort). **(A)** Rates of cardiovascular death by groups. **(B)** Rates of rehospitalization for heart failure by groups. **(C)** Rates of all-cause death by groups. **(D)** Rates of all-cause rehospitalization by groups.

In the validating cohorts, 360 (15.3%) patients died for cardiovascular reasons during 1-year follow-up. In the low, mid-low, mid-high, and high point groups, the cardiovascular mortalities were 3.8, 9.4, 19.4, and 26.3% (*P* < 0.0001), respectively. The unadjusted cumulative event rates for cardiovascular death by multi-biomarker groups were presented in Figure 2A.

**FIGURE 3:**
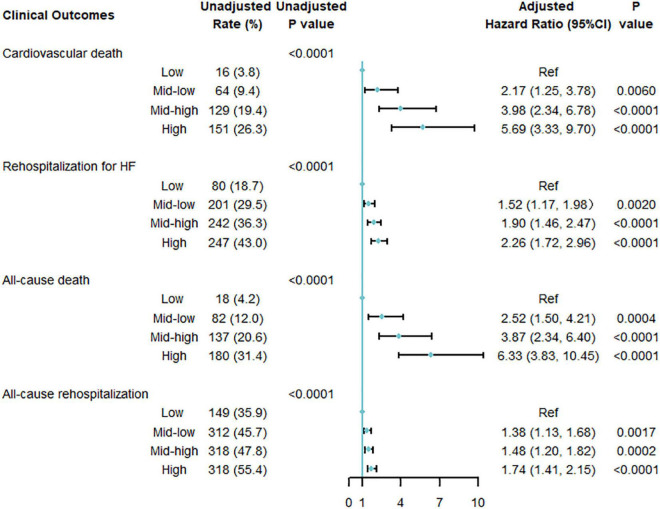
Multi-variable adjusted association between multi-biomarker group and 1-year clinical outcomes (validating cohort). Adjusted for age, sex, systolic blood pressure, heart rate, New York Heart Association classification, history of hypertension, atrial fibrillation, coronary heart disease, valvular heart disease, previous heart failure, current smoking [yes or no], heart failure phenotypes [HF with reduced ejection fraction (HFrEF), HF with mildly reduced ejection fraction (HfmrEF), or HF with preserved ejection fraction (HFpEF)], discharge use of angiotensin-converting enzyme inhibitors or angiotensin receptor blockers, β-blockers, diuretics, and aldosterone antagonists.

After multivariable adjustment, the cumulative risk of cardiovascular death increased in a graded fashion across multi-biomarker groups (Figure 3). With the low point group as the reference point, patients in the high point group had the highest risk of cardiovascular death (HR 5.69; 95% confidential interval [CI] 3.33, 9.70), followed by the mid-high point group (HR 3.98; 95% CI 2.34, 6.78) and mid-low point group (HR 2.17; 95% CI 1.25, 3.78). And the HR per 1-point increase was 1.14 (95% CI, 1.10, 1.18) (Supplementary Figure 4). The C-index of the model comprised of the biomarkers was 0.67 for cardiovascular death (Supplementary Table 10).

The multi-biomarker point pattern also stratified the risks of rehospitalization for HF, all-cause death, and all-cause rehospitalization during 1-year follow-up (Figures 2B–D), and patients with a higher multi-biomarker point were more prone to clinical outcomes (Figure 3 and Supplementary Figure 4). The C-indexes of the model were 0.60, 0.67, and 0.57 in predicting rehospitalization for HF, all-cause death, and all-cause rehospitalization, respectively (Supplementary Table 10).

### The Association Between Multi-Biomarker Point and Quality of Life

**FIGURE 4:**
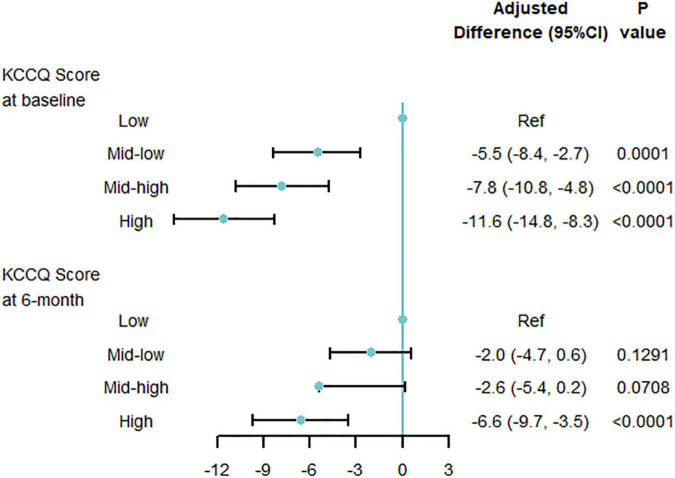
Quality of life by multi-biomarker point groups (validating cohort). KCCQ, Kansas City Cardiovascular Questionnaire; CI, confidential interval. Only including 3433 patients with KCCQ score at 6-month interview.

Patients with KCCQ-12 scores at baseline and 6-month interviews were included in this analysis. The mean ± SD of KCCQ score was 44.2 ± 23.0 at baseline, then significantly improved to 73.1 ± 20.8 at 6 months. At baseline, graded differences of quality of life were observed across multi-biomarker groups. Compared with low point group, mid-low point group (−5.5, 95% CI, −8.4 to −2.7, *P* = 0.0001), and mid-high point group (−7.8, 95% CI, −10.8 to −4.8, *P* < 0.0001), high-point group (−11.6, 95% CI, −14.8 to −8.3, *P* < 0.0001) had the lowest KCCQ-12 score, using the multi-variable generalized linear regression models. At the 6-month follow-up interview, the disparities were reduced but still significant (Figure 4). And the trends were similar when taking the multi-biomarker point as continuous variables (Supplementary Figure 5).

### Subgroup Analysis and Sensitivity Analysis

The associations between outcomes and multi-biomarker points were similar in patients with HFrEF, HFmrEF, or HFpEF (Supplementary Figures 6–9). In the sensitivity analysis, the relationship between multi-biomarker point and clinical outcomes was stable when using a competing risk model (Supplementary Figure 10), and the relationship between the quality of life and multi-biomarker point was similar in IPW analysis (Supplementary Figure 11). Furthermore, the results were still consistent when using β values to define the biomarker weights (Supplementary Tables 11, 12 and Supplementary Figures 12, 13).

## Discussion

**FIGURE 5:**
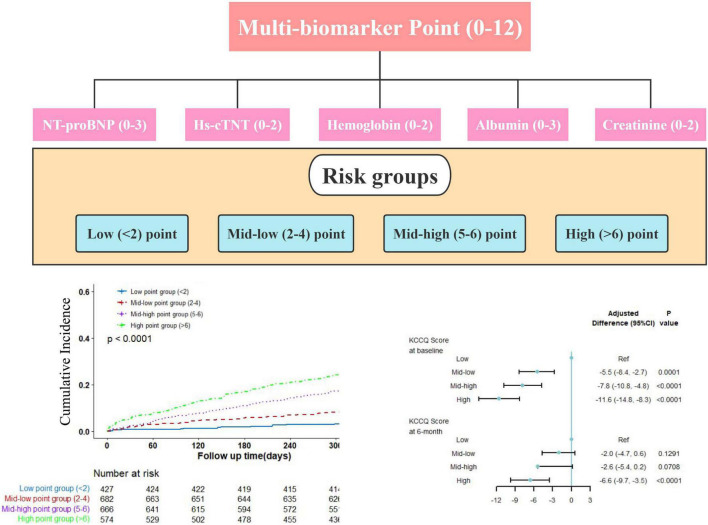
Central illustration. Patients were classified into low, mid-low, mid-high, and high point groups by baseline biomarkers. Increasing multi-biomarker points were associated with excess risk of cardiovascular mortality and worse quality of life.

In this large nationwide prospective cohort of patients hospitalized for HF, we have several important findings. Firstly, we revealed that systemic impairment reflected by abnormal values of conventional biomarkers was common. Secondly, patients with more biomarkers of abnormal value had a higher risk of clinical outcomes. Thirdly, the abnormal biomarker values were also associated with quality of life. Our analysis provided a new insight to depict the overall pattern of conventional biomarkers and the basic pathophysiological condition of patients, and quantitatively presented the cumulative effects of abnormal biomarkers both on clinical outcomes and quality of life (Figure 5).

Abnormal conventional biomarkers were common in patients hospitalized for HF, and our multi-biomarker point panel could reflect the overall burden of multiple basic pathophysiological states. To our knowledge, there has been no prior large study reporting the overall burden of abnormal conventional biomarkers with a weighted panel among patients hospitalized for HF. Most previous studies examined the distribution of individual biomarkers; for instance, the proportions of elevated NT-proBNP, hs-cTNT, and hsCRP were over two thirds (19), the rates of renal dysfunction were 40–50% (20), and the prevalence of DM, anemia, and dyslipidemia were 20, 26, and 50% (21), respectively. Several studies explored the prognostic values of various score systems in patients with acute HF: sequential organ function assessment score (SOFA) and ACUTE HF scores combined clinical, biomedical, and echocardiographic indexes to predict short- or long-term outcomes (22, 23); Norton score system, a tool to assess frailty had been studied in risk stratification (24); and albumin-bilirubin (ALBI) score reflecting liver function was also been discovered to associate with 1-year mortality (25). Unlike aforementioned score systems, our multi-biomarker point panel tried to integrate the information of multiple basic pathophysiological statuses and therefore reflected a patient’s overall basic condition and systemic impairment. Moreover, patients with HFrEF were more often to have higher multi-biomarker points than those with HFmrEF or HFpEF. The different multi-biomarker points across LVEF subgroups were mainly attributed to NT-proBNP and hs-cTNT (Supplementary Table 9). The lower levels of these two biomarkers indicated that patients with HFpEF were less likely to have myocardial stretch and cardiac injury, which is consistent with prior studies (26–28).

The multi-biomarker point is associated with long-term clinical outcomes. Patients with higher points at baseline were more vulnerable to death or rehospitalization after discharge. Unlike prior studies just focusing on the individual biomarker in isolation, such as NT-proBNP and hs-cTNT (3, 29, 30), our analysis quantitatively evaluated the composite relationship between clinical outcomes and multiple conventional biomarkers. The risk differences across patient groups might reflect that various pathophysiological states have cumulative adverse associations with long-term prognosis (17). From the perspectives of clinical practice and pathophysiology, our analysis included the most commonly used biomarkers which could present the overall basic condition of patients and have a significant relationship with mortality. The insights from our comprehensive multi-biomarker panel suggest that clinicians pay more attention to patients with higher points who are more prone to clinical outcomes and deliver personalized care to these patients rationally. Moreover, the predicting ability of the model comprised of the biomarkers is acceptable as well. Furthermore, the biomarker parameters are easy to obtain and calculate, which makes the point panel feasible in daily clinical practice.

To the best of our knowledge, our analysis was the first study focusing on the cumulative association with quality of life attributed to conventional multi-biomarkers among patients hospitalized for HF. The KCCQ score is an extensively validated scale in measuring the HF-specific quality of life (16, 31, 32). The increase of KCCQ score from the admission of index hospitalization to 6-month interview reflects the improvement of quality of life, which could be explained by the recovery from acute HF episode to a stable condition (33). Furthermore, we also found that patients with higher multi-biomarker points had worse quality of life both at admission and 6-month after discharge than those with lower points. This is reasonable because a higher point indicates a complex pathophysiological condition, which is usually accompanied by certain factors related to worse quality of life, such as comorbidities, therapeutic complexity, and financial burden (34). With our quantitative analysis of abnormal biomarker values, our multi-biomarker point could be considered as a novel, simple, and fast method to identify patients vulnerable to worse life quality in daily clinical practice.

Our multi-biomarker allowed physicians to quantitively evaluate the systemic impairment of various basic pathophysiological burdens, and it was a good predictor of post-discharge outcome among patients hospitalized for HF. It would be useful for physicians to focus on patients with higher points, tailor targeted therapy for specific pathophysiological disorders, and follow up the patients closer and more intensively.

This study has some limitations. First, because we aimed to present the overall pattern of these biomarkers and how the pattern relates to prognosis in a simple approach, we did not assess the severity of the pathophysiological state and its relationship with long-term prognosis. Our multi-biomarker points could only reflect the basic pathophysiological condition but not the severity of pathophysiological status for patients in HF episodes. Secondly, 14% of the surviving patients lacked the data of KCCQ scores at the 6-month interview. To minimize the potential bias, we performed an IPW analysis and obtained similar results. Thirdly, we only performed internal validation in our patient population; however, external validation would be necessary to further explore the reliability of the multi-biomarker point if another patient population is available. Finally, all enrolled patients were Chinese, and the generalizability of the conclusion to other populations should be with caution.

## Conclusion

Using this multi-biomarker point panel, we quantitatively described the overall pattern of basic pathophysiological burden reflected by abnormal biomarker values. The abnormal biomarkers had cumulative adverse associations with post-discharge clinical outcomes and quality of life. Our multi-biomarker point panel provided physicians with a simple and fast tool to assess systemic impairment and identify high-risk patients.

## Data Availability Statement

The original contributions presented in the study are included in the article/Supplementary Material, further inquiries can be directed to the corresponding author.

## Ethics Statement

The studies involving human participants were reviewed and approved by Fuwai Hospital Ethics Committees. The patients/participants provided their written informed consent to participate in this study.

## Author Contributions

GH contributed to the design, statistical analysis, and manuscript preparation of the study. QW contributed to the statistical analysis of the study. LZ, XHo, DH, XHa, JG, YW, and JL contributed to the revision of the manuscript. All authors contributed to the article and approved the submitted version.

## Conflict of Interest

JL reported receiving research grants, through Fuwai Hospital, from Chinese Government and Chinese Academy of Medical Sciences for work to improve the management of hypertension and blood lipids and to improve patient outcomes of cardiovascular disease and COVID-19; receiving research agreements, through the National Center for Cardiovascular Diseases and Fuwai Hospital, from Amgen for a multicenter clinical trial assessing the efficacy and safety of omecamtiv mecarbil and for dyslipidemic patient registration; receiving a research agreement, through Fuwai Hospital, from Sanofi for a multicenter clinical trial on the effects of sotagliflozin; receiving a research agreement, through Fuwai Hospital, with the University of Oxford for a multicenter clinical trial of empagliflozin; receiving a research agreement, through the National Center for Cardiovascular Diseases, from AstraZeneca for clinical research methods training outside the submitted work; and receiving a research agreement, through the National Center for Cardiovascular Diseases, from Lilly for physician training outside the submitted work. The remaining authors declare that the research was conducted in the absence of any commercial or financial relationships that could be construed as a potential conflict of interest.

## Publisher’s Note

All claims expressed in this article are solely those of the authors and do not necessarily represent those of their affiliated organizations, or those of the publisher, the editors and the reviewers. Any product that may be evaluated in this article, or claim that may be made by its manufacturer, is not guaranteed or endorsed by the publisher.
