# Supplementary material for: Multi-Biomarker Points and Outcomes in Patients Hospitalized for Heart Failure: Insights From the China PEACE Prospective Heart Failure Study
Source: Front Cardiovasc Med. 2022 Apr 7;9:835465. doi: 10.3389/fcvm.2022.835465 (PMC9021370; doi:10.3389/fcvm.2022.835465)
Supplement: Supplementary file 1 [file Data_Sheet_1.pdf]

SUPPLEMENTAL MATERIAL

TABLE OF CONTENTS

Figure S1 Study patient population development flow chart.

Table S1 Number of patients in each local collaborate center.

Data S1 Methods of biomarker assays.

Table S2 Number and missing rates of biomarker assays included in multi-biomarker panel.

Data S2 Adjudication criteria for cause of death.

Table S3 Association between biomarkers and cardiovascular death risk in developing cohort.

Table S4 Pearson's correlation coefficients between included biomarkers.

Table S5 Multi-biomarker weight point panel calculated using hazard ratios.

Table S6 Multi-biomarker points (calculated using hazard ratios) and subgroups.

Figure S2 Multi-biomarker point distribution in developing cohort.

Figure S3 Multi-biomarker point distribution in validating cohort.

Table S7 Baseline characteristics by multi-biomarker point groups in developing cohort.

Table S8 Baseline characteristics by multi-biomarker point groups in validating cohort.

Table S9 Biomarker distribution and multi-biomarker points by HF subgroups.

Figure S4 Clinical outcomes and multi-biomarker points (continuous variable; validating cohort).

Table S10 C-index of individual biomarker and multi-biomarker points on prognosis (validating cohort).

Figure S5 KCCQ scores and multi-biomarker points (continuous variables; validating cohort).

Figure S6 Subgroup analysis of clinical outcomes and multi-biomarker point(s) in heart failure with reduced ejection fraction (validating cohort).

Figure S7 Subgroup analysis of clinical outcomes and multi-biomarker point(s) in heart failure with mildly reduced ejection fraction (validating cohort).

Figure S8 Subgroup analysis of clinical outcomes and multi-biomarker point(s) in heart failure with preserved ejection fraction (validating cohort).

Figure S9 Subgroup analysis of KCCQ scores and multi-biomarker points (validating cohort).

Figure S10 Sensitivity analysis of multi-variable adjusted association between multi-biomarker point group and 1-year clinical outcomes (competing risk model; validating cohort).

Figure S11 Sensitivity analysis of effect of the burden of multi-biomarker point on quality of life (inverse probably weighting, IPW; validating cohort).

Table S11 Multi-biomarker weight point panel calculated using beta values.

Table S12 Multi-biomarker points (calculated using beta values) and subgroups.

Figure S12 Clinical outcomes and multi-biomarker points (calculated using beta values) subgroups (validating cohort).

Figure S13 KCCQ scores and multi-biomarker points (calculated using beta values) subgroups (validating cohort).

Figure S1. Study patient population development flow chart.

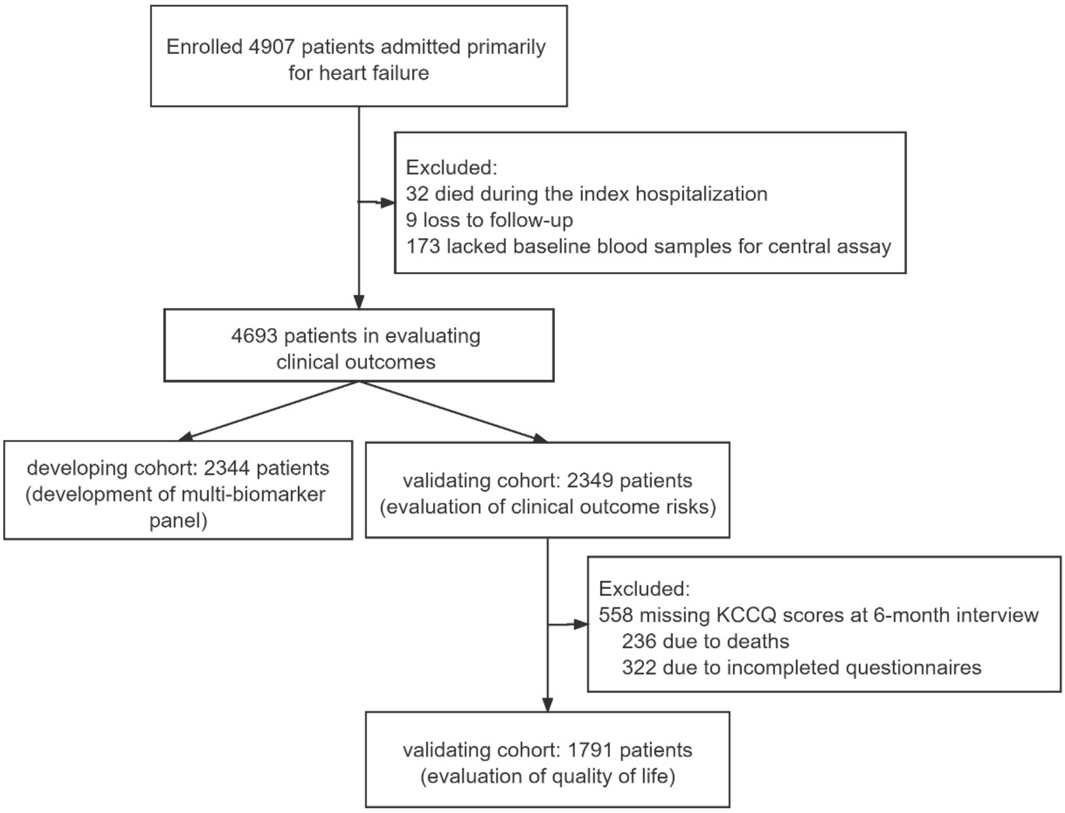

Table S1. Number of patients in each local collaborate center.

Local collaborate center	Number of patients
The First Affiliated Hospital of University of South China	388
Affiliated Hospital of Jining Medical University	384
Xinxiang Central Hospital	289
Hulunbeier People's Hospital	274
The Second Hospital of Shanxi Medical University	188
The First Affiliated Hospital of Xi'an Jiaotong University	186
Xinmin Peoples' Hospital	186
The Affiliated People Hospital of Inner Mongolia Medical University	181
Benxi Jinshan Hospital	169
The First Hospital of Jilin University	168
Affiliated Zhongshan Hospital of Dalian University	157
Shanxi Fenyang Hospital	145
Luoyang Dongfang Hospital	133
First Affiliated Hospital of Kunming Medical University	119
The Second Affiliated Hospital of Baotou Medical College	111
The First Affiliated Hospital of Zhengzhou University	109
Guangdong General Hospital	94
The First Affiliated Hospital of Hebei North University	91
Inner Mongolia People's Hospital	88
Fuwai Hospital	85

AVIC 363 Hospital	81
Qinghai Cardiovascular and Cerebrovascular Hospital	78
The Center Hospital of Maanshan	78
Qingdao Fuwai Hospital	74
The Second Affiliated Hospital of GZMU	73
Taizhou Hospital of Zhejiang Province	72
Qinyang People’s Hospital	61
Nanchong Central Hospital	54
Xiamen Cardiovascular Hospital Xiamen University	43
People's Government of Tibetan Autonomous Region	35
Shenyang the Fourth Hospital of People	35
The Second Affiliated Hospital of Xuzhou Medical University	34
Ningbo First Hospital	33
Brain Hospital of Hunan Province	32
Beijing Liangxiang Hospital	31
Beijing Chaoyang Hospital, Capital Medical University	31
Quwo County People's Hospital	31
Inner Mongolia Autonomous Region Hospital of Traditional Chinese Medicine	30
China-Japan Union Hospital of Jilin University	27
Central Hospital of Shenyang Sujiatun District	26
Baogang Hospital	22
The People's Hospital of Guangxi Zhuang Autonomous Region	22

Shenyang First People's Hospital	21
Xi’an No.1 Hospital	21
International Mongolia Hospital of Inner Mongolia	18
Puyang Oilfield General Hospital	15
The First Affiliated Hospital of Chongqing Medical University	15
Zhejiang Hospital	14
The Affiliated Yueqing Hospital of Wenzhou Medical University	13
The Affiliated Hospital of Xuzhou Medical University	12
Taiyuan City Central Hospital	9
Anshan Changda Hospital	7

Data S1. Methods of biomarker assays.

N-terminal pro-B type natriuretic peptide (NT-proBNP)

NT-proBNP was measured using the Roche E601. The limit of detection was 5 ng/L. The intra-assay coefficient of variation was ≤ 3.25%, and the inter-assay coefficient of variation was ≤ 8%.

High sensitivity Troponin T (hs-cTNT)

Hs-cTNT was measured using the Roche E601. The limit of detection was 3 ng/L. The intra-assay coefficient of variation was ≤ 8.15%, and the inter-assay coefficient of variation was ≤ 9.78%.

Creatinine

Creatinine was measured using the Beckman Coulter AU680. The limit of detection was 3.15 μmol/L. The intra-assay coefficient of variation was ≤ 3.75%, and the inter-assay coefficient of variation was ≤ 5%.

Alanine aminotransferase (ALT)

ALT was measured using the Beckman Coulter AU680. The limit of detection was 1 U/L. The intra-assay coefficient of variation was ≤ 5%, and the inter-assay coefficient of variation was ≤ 6.67%.

High sensitivity CRP (hsCRP)

HsCRP was measured using the Beckman Coulter AU680. The limit of detection was 0.02 mg/L. The intra-assay coefficient of variation was ≤ 6.21%, and the inter-assay coefficient of variation was ≤ 8.28%.

Glycosylated hemoglobin A_{1c} (HbA_{1c})

HbA_{1c} was measured using the Arkrey HA-8180. The intra-assay coefficient of variation was ≤ 3%, and the inter-assay coefficient of variation was ≤ 3%.

Total cholesterol (TC)

TC was measured using the Beckman Coulter AU680. The limit of detection was 0.07 mmol/L. The intra-assay coefficient of variation was ≤ 2.50%, and the inter-assay coefficient of variation was ≤ 3.33%.

Low-density lipoprotein cholesterol (LDL-C)

LDL-C was measured using the Beckman Coulter AU680. The limit of detection was 0.012 mmol/L. The intra-assay coefficient of variation was ≤ 3.97%, and the inter-assay coefficient of variation was ≤ 3.97%.

Triglycerides

Triglycerides was measured using the Beckman Coulter AU680. The limit of detection was 0.01 mmol/L. The intra-assay coefficient of variation was ≤ 6.25%, and the inter-assay coefficient of variation was ≤ 8.33%.

High-density lipoprotein cholesterol (HDL-C)

HDL-C was measured using the Beckman Coulter AU680. The limit of detection was 0.002 mmol/L. The intra-assay coefficient of variation was ≤ 7.50%, and the inter-assay coefficient of variation was ≤ 10%.

Table S2. Number and missing rate of biomarker assays included in multi-biomarker panel.

Biomarker	No. with data	No. Missing	Missing rate
NT-proBNP	4691	2	<0.1%
Hs-cTNT	4690	3	<0.1%
Hemoglobin	4526	137	2.9%
Albumin	4452	211	4.5%
Creatinine	4693	0	0%

Abbreviation: NT-proBNP: N-terminal pro-B type natriuretic peptide; Hs-cTNT: high-sensitivity troponin T.

Data S2. Adjudication criteria for cause of death

1. Cardiovascular death

Cardiovascular death includes death resulting from an acute myocardial infarction (MI), sudden cardiac death, death due to heart failure (HF) worsen, death due to stroke, and death due to other cardiovascular causes.

1) Acute Myocardial Infarction

The term MI should only be used when there is evidence of myocardial necrosis consistent with myocardial ischemia in an appropriate clinical context.

In general, the diagnosis of MI requires the following to be true:

	i. Cardiac necrosis	
	- Cardiac biomarkers	Cardiac biomarker results compatible with acute myocardial necrosis
	or - Autopsy	Myocardial necrosis/scar or coronary thrombus of an age consistent with the clinical presentation
AND	ii. Other supporting information:	
	- Presentation	i. Symptoms of ischemia; or<br>ii. Death
	or - ECG	i. Ischemic changes (not known to be old); or ii. Development of pathological Q waves
	or - Imaging	i. New myocardial defect; or<br>ii. Acute coronary occlusion

AND	iii. Exclusions:	No other likely diagnosis

2) Sudden Cardiac Death

Sudden death refers to meet the following circumstances:

• Witnessed death (i.e. participant seen at the time of death or within the preceding few days); and

• No evidence of an alternative cardiovascular or non-vascular cause of death (including no evidence to suggest Type 3 myocardial infarction)

3) Worsening Heart Failure

Heart failure is defined as the patient exhibits worsening symptoms of heart failure on presentation, has objective evidence of worsening of heart failure, and receives initiation or intensification of treatment specifically for heart failure.

4) Stroke

Stroke is defined as an acute symptomatic episode of focal or global neurological dysfunction caused by brain, spinal, or retinal vascular injury as a result of hemorrhage or infarction.

5) Other cardiovascular death

Death due to other cardiovascular disease includes deaths that are likely to be due to specific cardiovascular disorders (e.g. valvular heart disease, non-ischemic cardiomyopathy, primary arrhythmia, pulmonary embolism, primary pulmonary hypertension, ruptured aortic

aneurysm, acute limb ischemia), and deaths due to surgical and non-surgical investigations and procedures for other cardiovascular disease.

2. Non-cardiovascular death

Non-cardiovascular death was considered if an unequivocal and documented non-cardiovascular cause could be established as the primary cause of death (e.g., malignancy, infection, respiratory disease, liver disease,).

3. Presumed cardiovascular death / unknown death

Presumed cardiovascular death / unknown death was considered if there is no evidence of an alternative cardiovascular or non-vascular cause of death (including sudden cardiac death).

Table S3. Association between biomarkers and cardiovascular death risk in developing cohort.

	Adjusted HR	95% CI	p
**Included biomarkers**			
NT-proBNP ≥ 1400 ng/L	2.39	1.78, 3.22	<0.0001
Hs-cTNT > 14 ng/L	1.65	1.15, 2.38	0.0072
Anemia	1.42	1.09, 1.86	0.0100
Albumin ≤ 35 g/L	1.92	1.52, 2.43	<0.0001
Creatinine > 133 μmol/L	1.48	1.11, 1.98	0.0078
**Other biomarkers**			
ALT > 40 U/L	1.04	0.73, 1.49	0.8160
DM	1.19	0.91, 1.56	0.2099
HsCRP > 2 mg/L	1.08	0.81, 1.44	0.6085
Dyslipidemia	1.05	0.76, 1.46	0.7628
**Other candidate covariates**			
Demographics			
Age (per year)	1.004	0.99, 1.02	0.3991
Female	1.13	0.88, 1.45	0.3295
Vital sign			
SBP < 110 mmHg	Ref	Ref	Ref
SBP 110-129 mmHg	0.76	0.57, 1.02	0.0662
SBP ≥ 130 mmHg	0.55	0.40, 0.75	0.0001

HR < 80 b.p.m.	Ref	Ref	Ref
HR 80-99 b.p.m.	0.92	0.70, 1.20	0.5165
HR ≥ 100 b.p.m.	0.97	0.73, 1.31	0.8606
NYHA class			
NYHA class II	Ref	Ref	Ref
NYHA class III	1.30	0.82, 2.06	0.2712
NYHA class IV	1.54	0.97, 2.45	0.0695
Medical history & Risk factor			
Hypertension	0.61	0.47, 0.79	0.0001
Atrial fibrillation	1.20	0.94, 1.54	0.1428
Coronary heart diseases	1.14	0.88, 1.47	0.3150
Valvular heart diseases	0.77	1.04, 1.42	0.7874
Previous heart failure	1.26	0.96, 1.66	0.1023
Current smoking	0.87	0.65, 1.17	0.3698
Heart failure subgroup			
HFrEF	Ref	Ref	Ref
HFmrEF	0.99	0.73, 1.33	0.9350
HFpEF	0.82	0.61, 1.12	0.2138
Discharge medication			
ACEI/ARBs	0.98	0.76, 1.26	0.8433
β-blockers	0.80	0.61, 1.03	0.0864
Aldosterone antagonists	1.11	0.66, 1.85	0.6995

CCBs	0.87	0.50, 1.51	0.6276

Abbreviation: HR: hazard ratio; CI: confidential interval; NT-proBNP: N-terminal pro-B type natriuretic peptide; Hs-cTNT: high-sensitivity troponin T; Anemia: Hemoglobin < 120 g/L in men or hemoglobin < 110 g/L in women; DM: diabetes mellitus, glycosylated hemoglobin A_{1c} ≥ 6.5%; HsCRP: high-sensitivity C-reactive protein; SBP: systolic blood pressure; HR: heart rate; NYHA: New York Heart Association; HFrEF: heart failure with reduced ejection fraction; HFmrEF: heart failure with mildly reduced ejection fraction; HFpEF: heart failure with preserved ejection fraction; ACEI: angiotensin-converting enzyme inhibitor; ARB: angiotensin receptor blockers; CCB: calcium channel blocker.

Table S4. Pearson's correlation coefficients between included biomarkers.

	Hs-cTNT	Hemoglobin	Albumin	Creatinine
NT-proBNP	0.08	-0.18	-0.18	0.50
Hs-cTNT		-0.02	-0.01	0.06
Hemoglobin			0.26	-0.25
Albumin				-0.15

Abbreviation: NT-proBNP: N-terminal pro-B type natriuretic peptide; Hs-cTNT: high-sensitivity troponin T.

Note: Collinearity: Pearson's correlation coefficient >0.70.

Table S5. Multi-biomarker weight point panel calculated using hazard ratios.

Abnormal biomarker value(s)	Weight	Yes (√) or No (X)
NT-proBNP ≥ 1400 ng/L	3	
Hs-cTNT > 14 ng/L	2	
Anemia (hemoglobin < 120 g/L for male < 110 g/L for female)	2	
Albumin ≤ 35 g/L	3	
Creatinine > 133 μmol/L	2	
**Total points (√)**		

Abbreviation: NT-proBNP: N-terminal pro-B type natriuretic peptide; Hs-cTNT: high-sensitivity troponin T.

Note: Biomarker with lowest (anemia) hazard ratio (HR) value was defined as "2" weight, and the weights of other biomarkers were calculated by their HRs (keep round number). Each patient had a multi-biomarker point score ranges from 0 to 12 points.

Table S6. Multi-biomarker points (calculated using hazard ratios) and subgroups.

Multi-biomarker point(s)	Subgroups
<2 points	Low point
2-4 points	Mid-low point
5-6 points	Mid-high point
> 6 points	High point

Figure S2. Multi-biomarker point distribution in developing cohort.

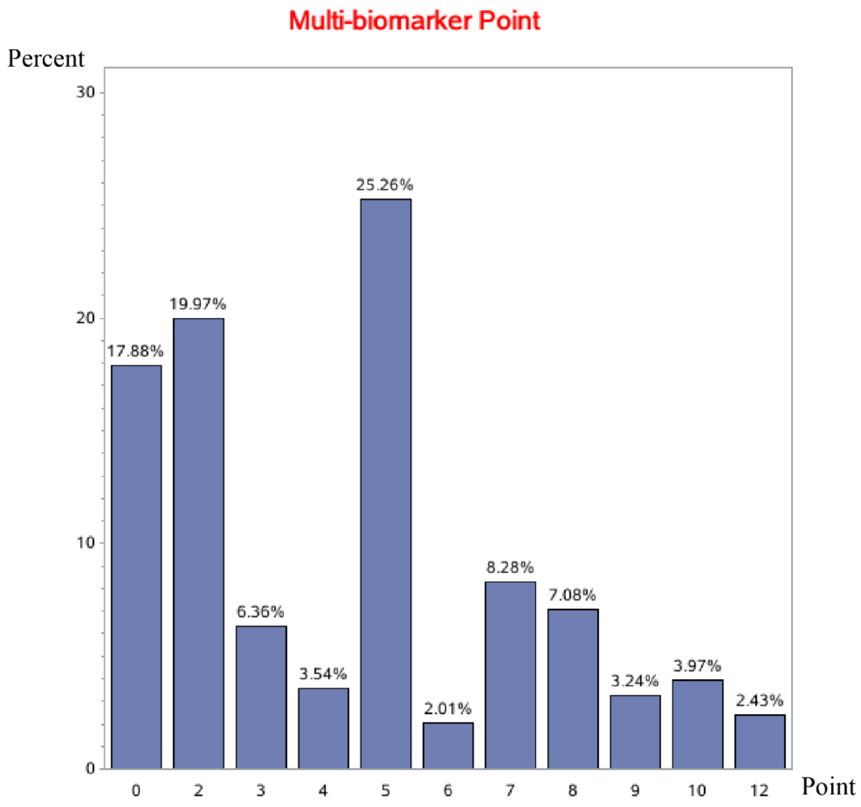

Figure S3. Multi-biomarker point distribution in validating cohort.

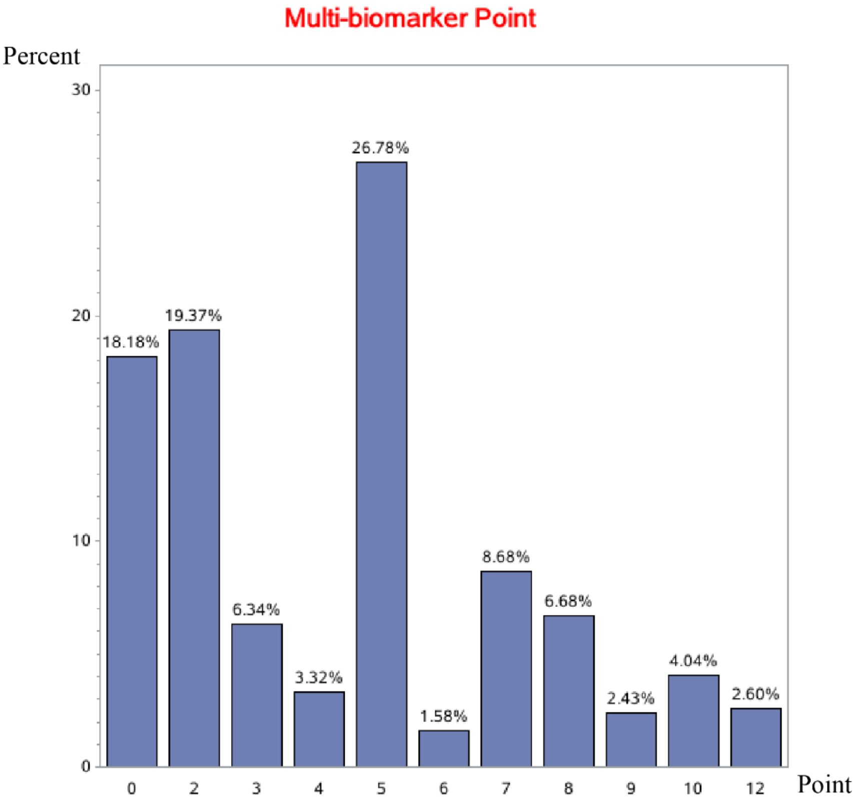

Table S7. Baseline characteristics by multi-biomarker point groups in developing cohort.

	Total (n=2344)	< 2 points (n=419)	2-4 points (n=700)	5-6 points (n=639)	> 6 points (n=586)	p value
**Demographic**						
Age, year, median (IQR)	67 (57, 76)	64 (55, 74)	66 (55, 75)	66 (57, 75)	71 (62, 79)	<0.0001
Female, n (%)	867 (37.0)	197 (47.0)	227 (32.4)	233 (36.5)	210 (35.8)	<0.0001
**Clinical characteristics**						
SBP, mmHg, median (IQR)	130 (117, 149)	130 (120, 147)	130 (119, 150)	130 (113, 145)	130 (114, 151)	0.1404
DBP, mmHg, median (IQR)	80 (70, 90)	80 (70, 90)	80 (70, 90)	80 (70, 90)	80 (68, 90)	0.0258
HR, b.p.m, median (IQR)	86 (74, 100)	80 (70, 96)	85 (74, 100)	90 (76, 105)	88 (76, 100)	<0.0001
NYHA class, n (%)						<0.0001
III	1032 (44.0)	196 (46.8)	318 (45.4)	272 (42.6)	246 (42.0)	
IV	961 (41.0)	99 (23.6)	254 (36.3)	306 (47.9)	302 (51.5)	
**Medical history & Risk factor**						

Hypertension, n (%)	1357 (57.9)	232 (55.4)	416 (59.4)	337 (52.7)	372 (63.5)	0.0010
Atrial fibrillation, n (%)	860 (36.7)	159 (38.0)	258 (36.9)	255 (39.9)	188 (32.1)	0.0368
CHD, n (%)	1370 (58.5)	231 (55.1)	415 (59.3)	347 (54.3)	377 (64.3)	0.0018
MI, n (%)	546 (23.3)	69 (16.5)	167 (23.9)	148 (23.2)	162 (27.7)	0.0006
VHD, n (%)	375 (16.0)	63 (15.0)	113 (16.2)	108 (16.9)	91 (15.5)	0.8534
Previous HF, n (%)	1646 (70.2)	263 (62.8)	484 (69.1)	469 (73.4)	430 (73.4)	0.0006
Stroke, n (%)	480 (20.5)	84 (20.1)	138 (19.7)	122 (19.1)	136 (23.2)	0.2916
COPD, n (%)	461 (19.7)	85 (20.3)	150 (21.4)	121 (18.9)	105 (17.9)	0.4190
Reduced renal function, n (%)	655 (27.9)	40 (9.6)	154 (22.0)	143 (22.4)	318 (54.3)	<0.0001
DM, n (%)	742 (31.7)	94 (22.4)	230 (32.9)	189 (29.6)	229 (39.1)	<0.0001
Current smoking, n (%)	581 (24.8)	106 (25.3)	192 (27.4)	166 (26.0)	117 (20.0)	0.0150
**Echocardiograph**						
LVEF, %, median (IQR)	43 (33, 56)	52 (39, 62)	44 (35, 56)	40 (30, 50)	43 (33, 54)	<0.0001
HF phenotype						<0.0001
HFrEF, n (%)	936 (39.9)	108 (25.8)	275 (39.3)	317 (49.6)	236 (40.3)	
HFmrEF, n (%)	541 (23.1)	80 (19.1)	162 (23.1)	152 (23.8)	147 (25.1)	

HFpEF, n (%)	867 (37.0)	231 (55.1)	263 (37.6)	170 (26.6)	203 (34.6)	
**In-hospital treatment**						
Coronary angiography	337 (14.4)	78 (18.6)	125 (17.9)	84 (13.2)	50 (8.5)	<0.0001
PCI	104 (4.4)	17 (4.1)	43 (6.1)	19 (3.0)	25 (4.3)	0.0418
**Treatment at Discharge**						
ACEI/ARBs, n (%)	1204 (51.4)	220 (52.5)	370 (52.9)	351 (54.9)	263 (44.9)	0.0030
β-blockers, n (%)	1399 (59.7)	270 (64.4)	421 (60.1)	388 (60.7)	320 (54.6)	0.0144
Aldosterone antagonists, n (%)	1483 (63.3)	243 (58.0)	419 (59.9)	454 (71.1)	367 (62.6)	<0.0001
CCBs, n (%)	348 (14.9)	73 (17.4)	107 (15.3)	60 (9.4)	108 (18.4)	<0.0001
Diuretics, n (%)	1598 (68.2)	254 (60.6)	451 (64.4)	477 (74.7)	416 (71.0)	<0.0001

Abbreviation: HF: heart failure; IQR: interquartile range; SBP: systolic blood pressure; DBP: diastolic blood pressure; HR: heart rate; NYHA class: New York Heart Association class; CHD: coronary heart disease; MI: myocardial infarction; VHD: valvular heart disease; HF: heart failure; COPD: chronic obstructive pulmonary disease; Reduced renal function: eGFR <60 ml/L/1.73m^2; DM: diabetes mellitus; LVEF: left ventricular ejection fraction; HFrEF: heart failure with reduced ejection fraction; HFmrEF: heart failure with mildly reduced ejection fraction; HFpEF: heart failure with preserved ejection fraction; PCI: percutaneous

coronary intervention; ACEI: angiotensin-converting enzyme inhibitor; ARB: angiotensin receptor blockers; CCB: calcium channel blocker.

Table S8. Baseline characteristics by multi-biomarker point groups in validating cohort.

	Total (n=2349)	< 2 points (n=427)	2-4 points (n=682)	5-6 points (n=666)	> 6 points (n=574)	p value
**Demographic**						
Age, year, median (IQR)	67 (57, 75)	64 (54, 72)	66 (56, 75)	67 (57, 75)	69 (60, 77)	<0.0001
Female, n (%)	896 (38.1)	216 (50.6)	244 (35.8)	247 (37.1)	189 (32.9)	<0.0001
**Clinical characteristics**						
SBP, mmHg, median (IQR)	130 (116, 148)	130 (119, 145)	130 (119, 150)	130 (111, 145)	132 (115, 152)	0.0067
DBP, mmHg, median (IQR)	80 (70, 90)	80 (70, 90)	80 (70, 90)	80 (70, 90)	80 (70, 90)	0.0708
HR, b.p.m, median (IQR)	86 (74, 101)	81 (71, 99)	85 (72, 100)	88 (75, 104)	88 (76, 100)	0.0001
NYHA class, n (%)						<0.0001
III	1044 (44.4)	218 (51.1)	307 (45.0)	294 (44.1)	225 (39.2)	
IV	971 (41.3)	88 (20.6)	253 (37.1)	314 (47.2)	316 (55.1)	
**Medical history & Risk factor**						

Hypertension, n (%)	1393 (59.3)	229 (53.6)	414 (60.7)	369 (55.4)	381 (66.4)	<0.0001
Atrial fibrillation, n (%)	852 (36.3)	174 (40.8)	243 (35.6)	246 (36.9)	189 (32.9)	0.0810
CHD, n (%)	1345 (57.3)	223 (52.2)	400 (58.7)	353 (53.0)	369 (64.3)	<0.0001
MI, n (%)	529 (22.5)	70 (16.4)	151 (22.1)	140 (21.0)	168 (29.3)	<0.0001
VHD, n (%)	384 (16.4)	75 (17.6)	100 (14.7)	123 (18.5)	86 (15.0)	0.1831
Previous HF, n (%)	1651 (70.3)	285 (66.7)	483 (70.8)	474 (71.2)	409 (71.3)	0.3669
Stroke, n (%)	483 (20.6)	82 (19.2)	136 (19.9)	129 (19.4)	136 (23.7)	0.1977
COPD, n (%)	467 (19.9)	87 (20.4)	142 (20.8)	135 (20.3)	103 (17.9)	0.6022
Reduced renal function, n (%)	690 (29.4)	36 (8.4)	135 (19.8)	172 (25.8)	347 (60.5)	<0.0001
DM, n (%)	742 (31.6)	114 (26.7)	199 (29.2)	197 (29.6)	232 (40.4)	<0.0001
Current smoking, n (%)	597 (25.4)	105 (24.6)	182 (26.7)	185 (27.8)	125 (21.8)	0.0820
**Echocardiograph**						
LVEF, %, median (IQR)	44 (34, 56)	50 (40, 61)	45 (35, 58)	40 (30, 50)	42 (33, 52)	<0.0001
HF phenotype						<0.0001
HFrEF, n (%)	890 (37.9)	98 (23.0)	234 (34.3)	318 (47.8)	240 (41.8)	
HFmrEF, n (%)	603 (25.7)	108 (25.3)	177 (26.0)	169 (25.4)	149 (26.0)	

HFpEF, n (%)	856 (36.4)	221 (51.8)	271 (39.7)	179 (26.9)	185 (32.2)	
**In-hospital treatment**						
Coronary angiography	294 (12.5)	58 (13.6)	94 (13.8)	88 (13.2)	54 (9.4)	0.0784
PCI	107 (4.6)	13 (3.0)	34 (5.0)	32 (4.8)	28 (4.9)	0.4293
**Treatment at Discharge**						
ACEI/ARBs, n (%)	1251 (53.3)	240 (56.2)	387 (56.7)	357 (53.6)	267 (46.5)	0.0016
β-blockers, n (%)	1369 (58.3)	258 (60.4)	417 (61.1)	379 (56.9)	315 (54.9)	0.0956
Aldosterone antagonists, n (%)	1490 (63.4)	245 (57.4)	435 (63.8)	455 (68.3)	355 (61.9)	0.0026
CCBs, n (%)	338 (14.4)	72 (16.9)	93 (13.6)	62 (9.3)	111 (19.3)	<0.0001
Diuretics, n (%)	1627 (69.3)	267 (62.5)	466 (68.3)	481 (72.2)	413 (72.0)	0.0028

Abbreviation: HF: heart failure; IQR: interquartile range; SBP: systolic blood pressure; DBP: diastolic blood pressure; HR: heart rate; NYHA class: New York Heart Association class; CHD: coronary heart disease; MI: myocardial infarction; VHD: valvular heart disease; HF: heart failure; COPD: chronic obstructive pulmonary disease; Reduced renal function: eGFR <60 ml/L/1.73m^2; DM: diabetes mellitus; LVEF: left ventricular ejection fraction; HFrEF: heart failure with reduced ejection fraction; HFmrEF: heart failure with mildly reduced ejection fraction; HFpEF: heart failure with preserved ejection fraction; PCI: percutaneous

coronary intervention; ACEI: angiotensin-converting enzyme inhibitor; ARB: angiotensin receptor blockers; CCB: calcium channel blocker.

Table S9. Biomarker distribution and multi-biomarker points by HF subgroups.

	Total (n=4693)	HFrEF (n=1826)	HFmrEF (n=1144)	HFpEF (n=1723)	p value
**Biomarkers**					
NT-proBNP, ng/L, median (IQR)	1474 (600, 3273)	2065 (963, 4288)	1594 (635, 3427)	929 (350, 2181)	<0.0001
NT-proBNP ≥ 1400ng/L, n (%)	2421 (51.6)	1159 (63.4)	619 (54.2)	643 (37.3)	<0.0001
Hs-cTNT, ng/L, median (IQR)	21.4 (12.7, 40.5)	24.4 (15.3, 43.2)	22.5 (13.0, 47.0)	17.8 (10.6, 33.3)	<0.0001
Hs-cTNT > 14ng/L, n (%)	3320 (70.7)	1435 (78.5)	821 (71.8)	1064 (61.8)	<0.0001
Hemoglobin, g/L, median (IQR)	134 (119, 148)	139 (125, 152)	132 (118, 146)	130 (114, 145)	<0.0001
Anemia, n (%)	877 (18.7)	236 (12.9)	239 (20.9)	402 (22.3)	<0.0001
Albumin, g/L, median (IQR)	38.8 (35.8, 41.7)	38.9 (35.8, 41.7)	38.8 (35.9, 41.6)	38.8 (35.6, 41.8)	0.9377
Albumin ≤ 35 g/L, n (%)	981 (20.9)	384 (21.0)	227 (19.9)	370 (21.5)	0.5750
Creatinine, μmol/L, median (IQR)	93 (78, 112)	95 (81, 113)	93 (78, 113)	90 (76, 109)	<0.0001

Creatinine > 133 µmol/L, n (%)	628 (13.4)	227 (12.4)	157 (13.7)	244 (14.2)	0.2907
Multi-biomarker point, median (IQR)	5 (2, 6)	5 (2, 7)	5 (2, 7)	3 (0, 5)	<0.0001
**Multi-biomarker point group**, n (%)					<0.0001
< 2 points	846 (18.0)	206 (11.3)	188 (16.4)	452 (26.2)	
2-4 points	1382 (29.5)	509 (27.9)	339 (29.6)	534 (31.0)	
5-6 points	1305 (27.8)	635 (34.8)	321 (28.1)	349 (20.3)	
≥ 7 points	1160 (24.7)	476 (26.1)	296 (25.9)	388 (22.5)	

Abbreviation: HF: heart failure; IQR: interquartile range; HFrEF: heart failure with reduced ejection fraction; HFmrEF: heart failure with mildly reduced ejection fraction; HFpEF: heart failure with preserved ejection fraction; NT-proBNP: N-terminal pro-B type natriuretic peptide; Hs-cTNT: high-sensitivity troponin T; Anemia: hemoglobin < 120 g/L in men or hemoglobin < 110 g/L in women.

Figure S4. Clinical outcomes and multi-biomarker points (continuous variable; validating cohort).

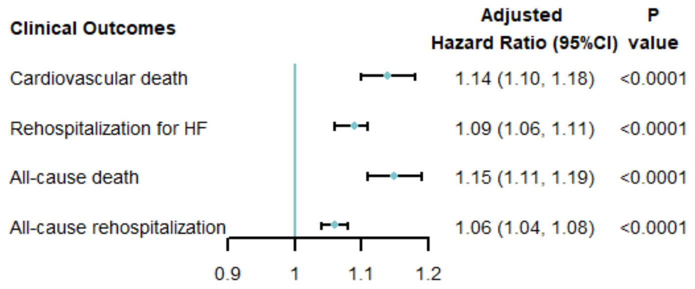

Abbreviation: CI: confidential interval.

Note: Adjusted for age, sex, systolic blood pressure, heart rate, New York Heart Association classification, history of hypertension, atrial fibrillation, coronary heart disease, valvular heart disease, previous heart failure, current smoking [yes or no], heart failure phenotypes [HFrEF, HFmrEF, HFpEF or missing LVEF], discharge use of angiotensin-converting enzyme inhibitor or angiotensin receptor blockers, β-blockers, diuretics, and aldosterone antagonists.

Table S10. C-index of individual biomarker and multi-biomarker points on prognosis (validating cohort).

	C-index
**Cardiovascular Death**	
Multi-biomarker point	0.67
NT-proBNP ≥ 1400 ng/L	0.63
Hs-cTNT > 14 ng/L	0.60
Anemia	0.52
Albumin ≤ 35 g/L	0.54
Creatinine > 133 μmol/L	0.54
**Rehospitalization for HF**	
Multi-biomarker point	0.60
NT-proBNP ≥ 1400 ng/L	0.57
Hs-cTNT > 14 ng/L	0.56
Anemia	0.54
Albumin ≤ 35 g/L	0.52
Creatinine > 133 μmol/L	0.54
**All-cause Death**	
Multi-biomarker point	0.67
NT-proBNP ≥ 1400 ng/L	0.62
Hs-cTNT > 14 ng/L	0.61
Anemia	0.54

Albumin ≤ 35 g/L	0.55
Creatinine > 133 μmol/L	0.55
**All-cause Rehospitalization**	
Multi-biomarker point	0.57
NT-proBNP ≥ 1400 ng/L	0.55
Hs-cTNT > 14 ng/L	0.54
Anemia	0.53
Albumin ≤ 35 g/L	0.51
Creatinine > 133 μmol/L	0.53

Abbreviation: NT-proBNP: N-terminal pro-B type natriuretic peptide; Hs-cTNT: high-sensitivity troponin T; Anemia: Hemoglobin < 120 g/L in men or hemoglobin < 110 g/L in women.

Figure S5. KCCQ scores and multi-biomarker points (continuous variables; validating cohort).

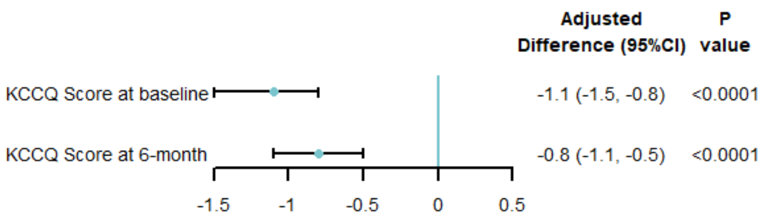

Abbreviation: CI: confidential interval.

Note: Adjusted for age, sex, systolic blood pressure, heart rate, New York Heart Association classification, history of hypertension, atrial fibrillation, coronary heart disease, valvular heart disease, previous heart failure, current smoking [yes or no], heart failure phenotypes [HFrEF, HFmrEF, HFpEF or missing LVEF], discharge use of angiotensin-converting enzyme inhibitor or angiotensin receptor blockers, β-blockers, diuretics, and aldosterone antagonists.

Figure S6. Subgroup analysis of clinical outcomes and multi-biomarker point(s) in heart failure with reduced ejection fraction (validating cohort).

Clinical Outcomes (HFrEF)		Unadjusted Rate (%)	Unadjusted P value	Adjusted Hazard Ratio (95%CI)	P value
Cardiovascular death			<0.0001		
	Low	5 (5.1)			
	Mid-low	21 (9.0)		Ref	
	Mid-high	65 (20.4)		2.17 (1.32, 3.57)	0.0023
	High	74 (30.8)		3.49 (2.12, 5.74)	<0.0001
Rehospitalization for HF			0.0260		
	Low	21 (21.4)		Ref	
	Mid-low	78 (33.3)		1.42 (0.86, 2.34)	0.1708
	Mid-high	113 (35.5)		1.53 (0.94, 2.49)	0.0860
	High	92 (38.3)		1.56 (0.94, 2.59)	0.0878
All-cause death			<0.0001		
	Low	5 (5.1)			
	Mid-low	27 (11.5)		Ref	
	Mid-high	68 (21.4)		1.76 (1.12, 2.76)	0.0145
	High	81 (33.8)		3.00 (1.92, 4.71)	<0.0001
All-cause rehospitalization			0.1479		
	Low	35 (35.7)		Ref	
	Mid-low	105 (44.9)		1.18 (0.79, 1.76)	0.4107
	Mid-high	138 (43.4)		1.21 (0.81, 1.79)	0.3521
	High	118 (48.2)		1.29 (0.85, 1.95)	0.2279

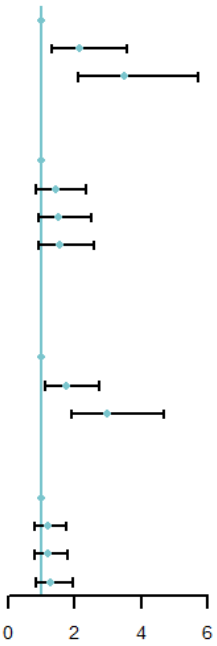

Abbreviation: CI: confidential interval.

Note: Adjusted for age, sex, systolic blood pressure, heart rate, New York Heart Association classification, history of hypertension, atrial fibrillation, coronary heart disease, valvular heart disease, previous heart failure, current smoking [yes or no], discharge use of angiotensin-converting enzyme inhibitor or angiotensin receptor blockers, β-blockers, diuretics, and aldosterone antagonists.

Figure S7. Subgroup analysis of clinical outcomes and multi-biomarker point(s) in heart failure with mildly reduced ejection fraction (validating cohort).

Clinical Outcomes (HFmrEF)		Unadjusted Rate (%)	Unadjusted P value	Adjusted Hazard Ratio (95%CI)	P value
Cardiovascular death			<0.0001		
	Low	3 (2.8)			
	Mid-low	18 (10.2)		Ref	
	Mid-high	34 (20.1)		1.62 (0.90, 2.90)	0.1068
	High	39 (26.2)		2.10 (1.17, 3.77)	0.0132
Rehospitalization for HF			<0.0001		
	Low	25 (23.2)		Ref	
	Mid-low	50 (28.3)		1.16 (0.71, 1.88)	0.5617
	Mid-high	63 (37.3)		1.50 (0.93, 2.42)	0.0959
	High	71 (47.7)		1.92 (1.18, 3.13)	0.0091
All-cause death			<0.0001		
	Low	3 (2.8)			
	Mid-low	19 (10.7)		Ref	
	Mid-high	36 (21.3)		1.67 (0.95, 2.96)	0.0755
	High	50 (33.6)		2.81 (1.61, 4.91)	0.0003
All-cause rehospitalization			0.0004		
	Low	39 (36.1)		Ref	
	Mid-low	84 (47.5)		1.40 (0.96, 2.06)	0.0848
	Mid-high	87 (51.5)		1.43 (0.97, 2.11)	0.0742
	High	93 (62.4)		1.81 (1.21, 2.70)	0.0037

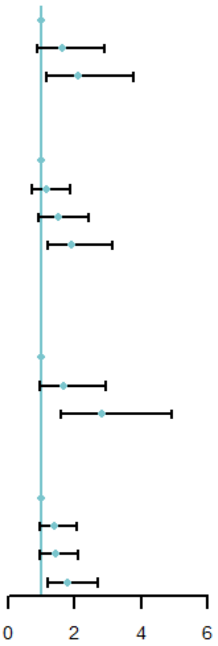

Abbreviation: CI: confidential interval.

Note: Adjusted for age, sex, systolic blood pressure, heart rate, New York Heart Association classification, history of hypertension, atrial fibrillation, coronary heart disease, valvular heart disease, previous heart failure, current smoking [yes or no], discharge use of angiotensin-converting enzyme inhibitor or angiotensin receptor blockers, β-blockers, diuretics, and aldosterone antagonists.

Figure S8. Subgroup analysis of clinical outcomes and multi-biomarker point(s) in heart failure with preserved ejection fraction (validating cohort).

Clinical Outcomes (HFpEF)		Unadjusted Rate (%)	Unadjusted P value	Adjusted Hazard Ratio (95%CI)	P value
Cardiovascular death			<0.0001		
	Low	8 (3.6)			
	Mid-low	25 (9.2)		Ref	
	Mid-high	30 (16.8)		1.51 (0.86, 2.63)	0.1484
	High	38 (20.5)		1.90 (1.13, 3.20)	0.0159
Rehospitalization for HF			<0.0001		
	Low	34 (15.4)		Ref	
	Mid-low	73 (26.9)		1.73 (1.14, 2.64)	0.0102
	Mid-high	66 (36.9)		2.64 (1.71, 4.07)	<0.0001
	High	84 (45.4)		3.39 (2.20, 5.23)	<0.0001
All-cause death			<0.0001		
	Low	10 (4.5)			
	Mid-low	36 (13.3)		Ref	
	Mid-high	33 (18.4)		1.15 (0.70, 1.88)	0.5912
	High	49 (26.5)		1.76 (1.12, 2.75)	0.0134
All-cause rehospitalization			<0.0001		
	Low	75 (33.9)		Ref	
	Mid-low	123 (45.4)		1.38 (1.03, 1.96)	0.0339
	Mid-high	93 (52.0)		1.80 (1.30, 2.48)	0.0004
	High	107 (57.8)		2.03 (1.47, 2.81)	<0.0001

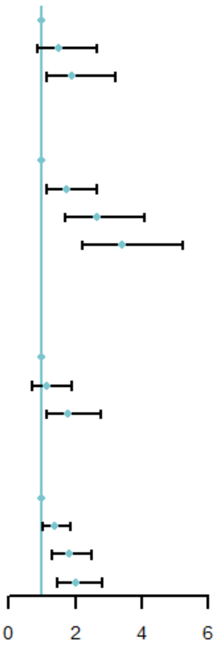

Abbreviation: CI: confidential interval.

Note: Adjusted for age, sex, systolic blood pressure, heart rate, New York Heart Association classification, history of hypertension, atrial fibrillation, coronary heart disease, valvular heart disease, previous heart failure, current smoking [yes or no], discharge use of angiotensin-converting enzyme inhibitor or angiotensin receptor blockers, β-blockers, diuretics, and aldosterone antagonists.

Figure S9. Subgroup analysis of KCCQ scores and multi-biomarker points (validating cohort).

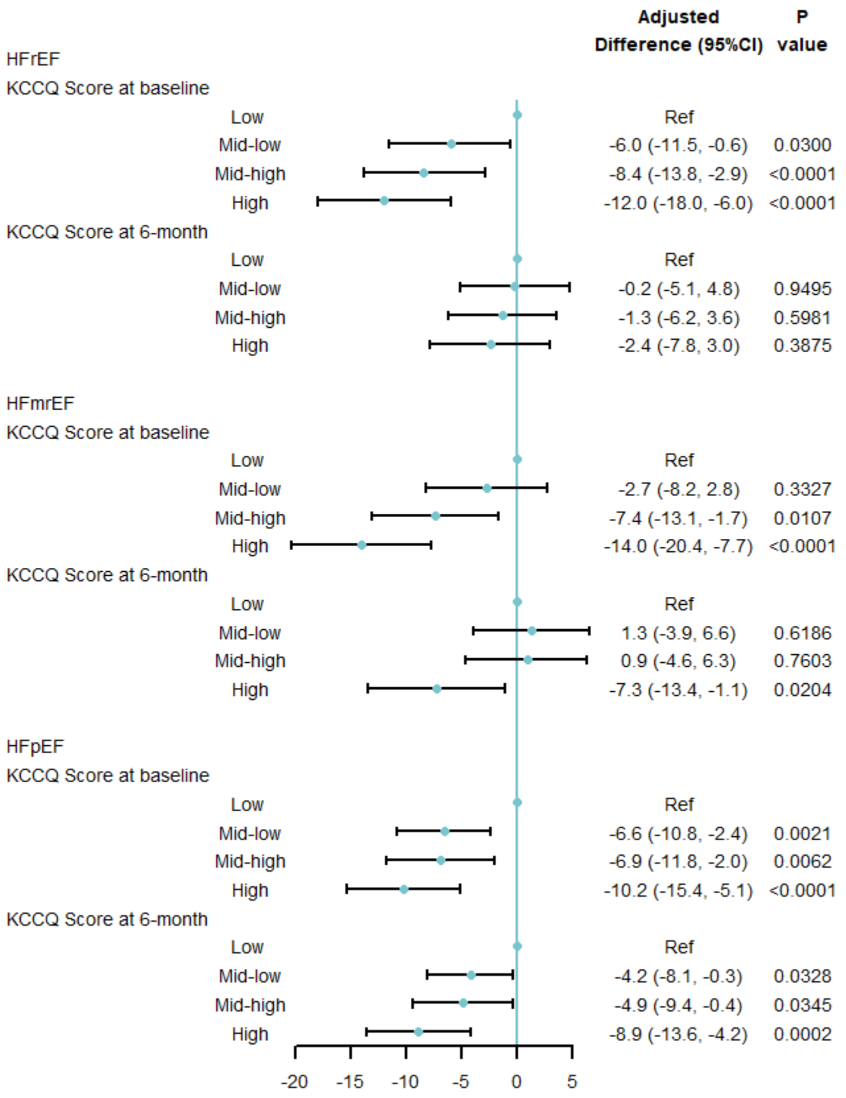

Abbreviation: CI: confidential interval.

Note: Adjusted for age, sex, systolic blood pressure, heart rate, New York Heart Association classification, history of hypertension, atrial fibrillation, coronary heart disease, valvular heart disease, previous heart failure, current smoking [yes or no], discharge use of angiotensin-converting enzyme inhibitor or angiotensin receptor blockers, β-blockers, diuretics, and aldosterone antagonists.

Figure S10. Sensitivity analysis of multi-variable adjusted association between multi-biomarker point group and 1-year clinical outcomes (competing risk model; validating cohort).

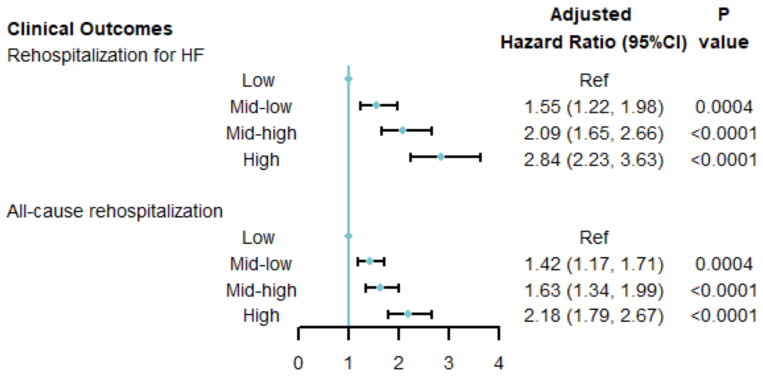

Abbreviation: CI: confidential interval.

Note: Adjusted for age, sex, systolic blood pressure, heart rate, New York Heart Association classification, history of hypertension, atrial fibrillation, coronary heart disease, valvular heart disease, previous heart failure, current smoking [yes or no], heart failure phenotypes [HFrEF, HFmrEF, HFpEF or missing LVEF], discharge use of angiotensin-converting enzyme inhibitor or angiotensin receptor blockers, β-blockers, diuretics, and aldosterone antagonists.

Figure S11. Sensitivity analysis of effect of the burden of multi-biomarker point on quality of life (inverse probably weighting, IPW; validating cohort).

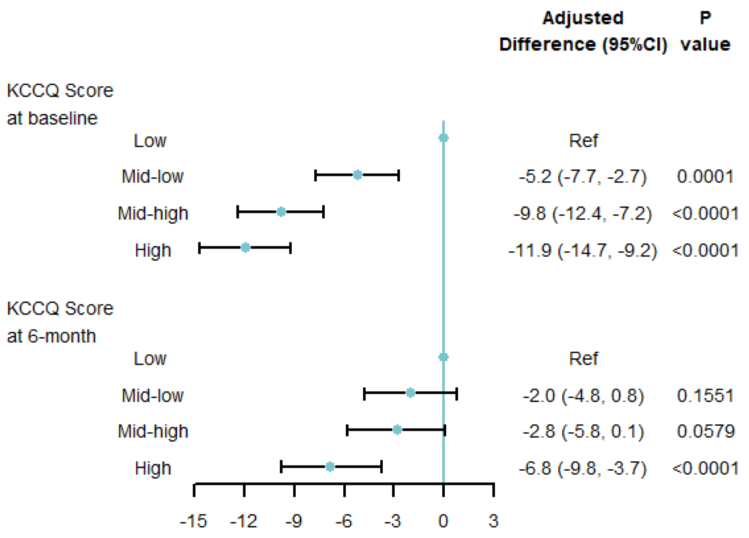

Abbreviation: CI: confidential interval.

Note: Adjusted for age, sex, systolic blood pressure, heart rate, New York Heart Association classification, history of hypertension, atrial fibrillation, coronary heart disease, valvular heart disease, previous heart failure, current smoking [yes or no], heart failure phenotypes [HFrEF, HFmrEF, HFpEF or missing LVEF], discharge use of angiotensin-converting enzyme inhibitor or angiotensin receptor blockers, β-blockers, diuretics, and aldosterone antagonists.

Table S11. Multi-biomarker weight point panel calculated using beta values.

Abnormal biomarker value(s)	Weight	Yes (√) or No (X)
NT-proBNP ≥ 1400 ng/L	2	
Hs-cTNT > 14 ng/L	1	
Anemia (hemoglobin < 120 g/L for male < 110 g/L for female)	1	
Albumin ≤ 35 g/L	2	
Creatinine > 133 μmol/L	1	
**Total points (√)**		

Abbreviation: NT-proBNP: N-terminal pro-B type natriuretic peptide; Hs-cTNT: high-sensitivity troponin T.

Note: Biomarker with lowest (anemia) beta value was defined as "1" weight, and the weights of other biomarkers were calculated by their beta values (keep round number). Each patient had a multi-biomarker point score ranges from 0 to 7 points.

Table S12. Multi-biomarker points (calculated using beta values) and subgroups.

Multi-biomarker point(s)	Subgroups	Number of patients (%)
< points	Low point	846 (18.0)
2-4 points	Mid-low point	1382 (29.4)
5-6 points	Mid-high point	1258 (26.8)
> 6 points	High point	1207 (25.7)

Figure S12. Clinical outcomes and multi-biomarker points (calculated using beta values) subgroups. (validating cohort)

Clinical Outcomes		Unadjusted Rate (%)	Unadjusted P value	Adjusted Hazard Ratio (95%CI)	P value
Cardiovascular death			<0.0001		
	Low	16 (3.8)		Ref	
	Mid-low	64 (9.4)		2.16 (1.24, 3.76)	0.0063
	Mid-high	128 (19.9)		4.08 (2.39, 6.94)	<0.0001
	High	152 (25.5)		5.45 (3.20, 9.28)	<0.0001
Rehospitalization for HF			<0.0001		
	Low	80 (18.7)		Ref	
	Mid-low	201 (29.5)		1.51 (1.16, 1.97)	0.0021
	Mid-high	240 (37.3)		1.96 (1.50, 2.55)	<0.0001
	High	249 (41.8)		2.15 (1.64, 2.82)	<0.0001
All-cause death			<0.0001		
	Low	18 (4.2)		Ref	
	Mid-low	82 (12.0)		2.50 (1.50, 4.19)	0.0005
	Mid-high	136 (21.2)		3.96 (2.40, 6.55)	<0.0001
	High	181 (30.4)		6.04 (3.66, 9.96)	<0.0001
All-cause rehospitalization			0.1479		
	Low	149 (34.9)		Ref	
	Mid-low	312 (45.8)		1.37 (1.12, 1.68)	0.0019
	Mid-high	315 (48.9)		1.53 (1.24, 1.88)	<0.0001
	High	321 (53.9)		1.66 (1.35, 2.05)	<0.0001

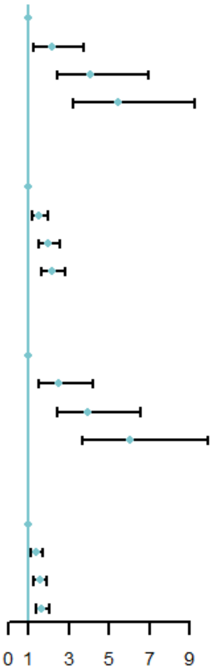

Abbreviation: CI: confidential interval.

Note: Adjusted for age, sex, systolic blood pressure, heart rate, New York Heart Association classification, history of hypertension, atrial fibrillation, coronary heart disease, valvular heart disease, previous heart failure, current smoking [yes or no], heart failure phenotypes [HFrEF, HFmrEF, HFpEF or missing LVEF], discharge use of angiotensin-converting enzyme inhibitor or angiotensin receptor blockers, β-blockers, diuretics, and aldosterone antagonists.

Figure S13. KCCQ scores and multi-biomarker points (calculated using beta values) subgroups. (validating cohort)

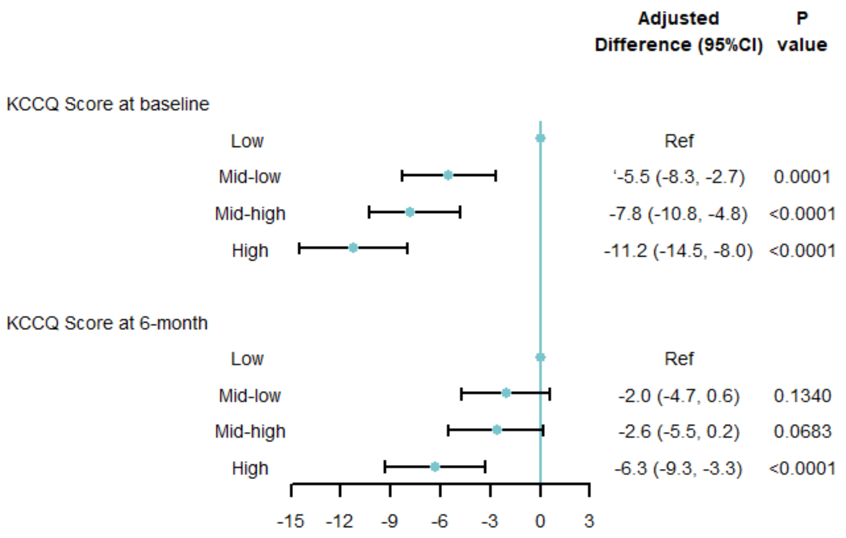

Abbreviation: CI: confidential interval.

Note: Adjusted for age, sex, systolic blood pressure, heart rate, New York Heart Association classification, history of hypertension, atrial fibrillation, coronary heart disease, valvular heart disease, previous heart failure, current smoking [yes or no], heart failure phenotypes [HFrEF, HFmrEF, HFpEF or missing LVEF], discharge use of angiotensin-converting enzyme inhibitor or angiotensin receptor blockers, β-blockers, diuretics, and aldosterone antagonists.
